# The association between depressive symptoms or depression and health outcomes in adults with low back pain with or without radiculopathy: protocol of a systematic review

**DOI:** 10.1186/s13643-019-1192-4

**Published:** 2019-11-08

**Authors:** Jessica J. Wong, Andrea C. Tricco, Pierre Côté, Laura C. Rosella

**Affiliations:** 1 Epidemiology Division, Dalla Lana School of Public Health, University of Toronto, 155 College Street, 6th floor, Toronto, ON M5T 3M7 Canada; 2 Centre for Disability Prevention and Rehabilitation, Ontario Tech University and Canadian Memorial Chiropractic College, 2000 Simcoe Street North, Oshawa, ON L1H 7K4 Canada; 3 Li Ka Shing Knowledge Institute, St. Michael’s Hospital, Knowledge Translation Program, 209 Victoria Street, East Building, Room 716, Toronto, ON M5B 1W8 Canada; 4 Institute of Health Policy, Management and Evaluation, University of Toronto, 155 College Street, 4th floor, Toronto, ON M5T 3M7 Canada; 5 Faculty of Health Sciences, Ontario Tech University, 2000 Simcoe Street North, Oshawa, ON L1H 7K4 Canada; 6 ICES, 155 College Street, Toronto, ON M5T 3M7 Canada

**Keywords:** Systematic review, Knowledge synthesis, Low back pain, Depression, Depressive symptoms

## Abstract

**Background:**

A considerable proportion of adults with low back pain (LBP) suffer from depressive symptoms or depression. Those with depressive symptoms or depression may be at risk of poorer LBP recovery and require more health care. Understanding the role of prognostic factors for LBP is critically important to guide management and health services delivery. Our objective is to conduct a systematic review to assess the association between depressive symptoms or depression and health outcomes in adults with LBP with or without radiculopathy.

**Methods:**

Electronic databases including MEDLINE, Embase, CINAHL, and PsycINFO will be searched from inception to April 2019 to identify relevant studies. Additional citations will be identified by searching reference lists of included studies and related systematic reviews. Cohort and case-control studies assessing the association between depressive symptoms/depression and health outcomes in adults aged 16 years and older with LBP with or without radiculopathy will be included. The following will be included: depressive symptoms as measured on standardized questionnaires (e.g., Center for Epidemiologic Studies Depression Scale, Beck Depression Index), and depression as standardized diagnoses (e.g., International Classification of Diseases codes) or self-reported depression diagnosis on standardized questionnaires. Outcomes of interest are standardized measures for pain, disability, overall health status, satisfaction with care, and health care utilization. These are informed by core outcome domains that international expert panels consider important for LBP research. Pairs of reviewers will screen articles retrieved from the search, extract data, and assess risk of bias using the Risk Of Bias In Non-randomized Studies-of Exposures (ROBINS-E) tool. Reviewers will use these criteria to inform their judgment on the internal validity of studies (e.g., low, moderate, or high risk of bias). If studies are deemed homogeneous, a random effects meta-analysis on the association between depressive symptoms and health outcomes will be performed. The results of the included studies will be descriptively outlined if studies are deemed heterogeneous.

**Discussion:**

The impact of depressive symptoms and depression on health- and health care-related outcomes for LBP with or without radiculopathy will be assessed and quantified. Findings of this systematic review will advance our understanding of LBP prognosis, and guide decision-making and improve quality of care for adults with LBP.

**Systematic review registration:**

PROSPERO CRD42019130047

## Background

Low back pain (LBP) is characterized by pain in the region between the costal margin and inferior gluteal folds [1] and may present with radiculopathy (involvement of the spinal nerve roots). LBP is a common condition that is burdensome to patients and health systems. Approximately 80% of people suffer from at least one episode of LBP during their lifetime, which may be traumatic (e.g., traffic or occupational injuries) or non-traumatic in nature [2, 3]. Although most episodes resolve, 10 to 20% of adults with LBP experience chronic symptoms, functional limitations, or difficulties returning to work [4, 5]. Importantly, LBP is the leading cause of years lived with disability globally [2, 6, 7] and is associated with high health care use and costs [8–14].

A considerable proportion of adults with LBP experience depressive symptoms or depression, which have the potential to negatively impact health outcomes and health care use. LBP and major depressive disorder are prevalent conditions and are both among the top five leading causes of years lived with disability globally [10, 11, 15–17]. It is estimated that 20 to 25% of adults with LBP also experience depressive symptoms or depression [18–20] and may be at risk of poorer recovery from LBP or more health care utilization. Specifically, some studies suggest that patients with depressive symptoms are more likely to have higher pain intensity, greater disability, and poorer quality of life, work outcomes and overall recovery related to LBP [21–28]. Patients with both LBP and depressive symptoms or depression appear to seek more health care and have poorer treatment outcomes [29, 30].

Given the prevalence of both conditions and concerns around the role of depressive symptoms on LBP recovery, it is critically important to understand the association between depressive symptoms/depression and health outcomes in adults with LBP. To our best knowledge, a systematic review examining the impact of depressive symptoms and depression on health care utilization in adults with LBP has not been previously conducted. Previous systematic reviews examining depressive symptoms and depression as prognostic factors affecting clinical or work-related outcomes for LBP require updating, as literature searches were completed before or up to early 2016 [22, 31–36]. Of these, the reviews with most recent literature searches were conducted by Alhowimel et al. (up to early 2016) [35] and by Pinheiro et al. (up to 2014) [22]. Alhowimel et al. targeted adults with chronic LBP (≥ 12 weeks’ duration) who received physiotherapy interventions and excluded those with spinal stenosis [35]. Pinheiro et al. targeted adults with acute/subacute LBP (≤ 12 weeks’ duration) and excluded those with sciatica and spinal stenosis [22]. Findings of these two reviews are therefore limited in generalizability to these LBP subgroups. Moreover, many primary studies have been published in this area since 2014 [37–47], particularly around disability, medication use, and surgical outcomes. A comprehensive and up-to-date systematic review is needed to inform future research and practice, and improve health services delivery and quality of care for LBP.

Our objective is to conduct a systematic review to assess the association between depressive symptoms or depression and health outcomes (i.e., pain, disability, overall health status, satisfaction with care, and health care utilization) in adults with LBP with or without radiculopathy.

## Methods

### Protocol

This systematic review protocol was developed using the Preferred Reporting Items for Systematic Review and Meta-Analysis Protocols (PRISMA-P) [48] to guide the reporting of the protocol (see Additional file 1). The systematic review will be reported based on the Preferred Reporting Items for Systematic Reviews and Meta-Analyses (PRISMA) statement [49]. The systematic review protocol has been registered with the International Prospective Register of Systematic Reviews (PROSPERO) database (CRD42019130047) [50].

### Eligibility criteria

#### Population

Our systematic review will target studies of adults aged 16 years or older with LBP with or without radiculopathy. LBP is defined as pain localized below the costal margin and above the inferior gluteal folds with or without referred leg pain, in the absence of any underlying serious or major pathology [1]. Radiculopathy refers to inflammation, injury, or compression of the spinal nerve roots that can present as pain, weakness, or numbness in a myotomal or dermatomal distribution. Lumbar radiculopathy may be attributed to spinal stenosis (narrowing of the spinal canal) or lumbar disk herniation (localized displacement of disk material beyond the normal margins of the intervertebral disk space) [51, 52]. Studies of LBP due to major structural or serious pathology will be excluded, such as spinal fractures, spinal dislocations, spinal cord injury, inflammatory arthritides, neoplasms, or malignancies. Studies targeting LBP with or without referred leg pain or radiculopathy will be eligible, as described with terms including mechanical LBP, lumbago, lumbar sprain or strain, lumbopelvic pain, lumbar radiculopathy, lumbar disk herniation, sacroiliac syndrome, sciatica, and spinal stenosis. Surgical populations will also be included, such as adults who had lumbar fusion, discectomy, laminectomy, or decompression. Studies with mixed populations such as adolescents and adults will be included if the results are stratified for adults aged 16 years and older.

#### Exposure

Studies that assess depressive symptoms or depression as the exposure will be included. Depressive symptoms as self-reported symptoms of depression on standardized questionnaires (e.g., Center for Epidemiologic Studies Depression Scale, Beck Depression Index) will be assessed. Diagnosed depression has two main categories: major depressive disorder/episode and dysthymia [53]. Major depressive disorder/episode presents with symptoms such as depressed mood, loss of interest and enjoyment, and decreased energy, and can be categorized as mild, moderate, or severe based on the symptom frequency and severity [53]. Dysthymia is a persistent or chronic form of mild depression with symptoms similar to depressive episodes but are less intense and persist longer [53]. Studies will be classified as targeting major depressive disorder/episodes or dysthymia based on the use of these terms. Studies where the depression diagnosis is self-reported on standardized questionnaires will also be included.

#### Comparators

Depressive symptoms or depression compared to the absence of depressive symptoms or depression will be examined. Higher severity of depressive symptoms or depression compared to lower severity will also be examined based on, respectively, scoring of standardized questionnaires (e.g., severe versus mild depressive symptoms using standardized thresholds on the Beck Depression Index) and standardized diagnoses (e.g., severe depressive episode versus mild depressive episode using the International Classification of Diseases (ICD) codes). Based on previous literature in LBP populations, the following clinical cut-points on depressive questionnaires will be considered homogeneous: ≥ 16 points on Center for Epidemiologic Studies Depression Scale, ≥ 15 on Beck Depression Index, ≥ 10 on Patient Health Questionnaire, and ≥ 8 on Depression Scale of the Hospital Anxiety and Depression Scale [28, 54–56].

#### Outcomes

The following health outcomes will be targeted: (1) pain (e.g., pain intensity), (2) disability (e.g., impairment, activity limitations, participation restriction), (3) overall health status (e.g., health-related quality of life, recovery), (4) satisfaction with care, and (5) health care utilization (e.g., physician visits, emergency department visits, hospitalizations, spinal imaging). These are informed by core outcome domains that are considered important for LBP research among international panels of experts [57–59]. Only standardized outcome measures such as standardized questionnaires or administrative data for the aforementioned health outcomes will be included. Questionnaires for health outcomes include: (1) Visual Analog Scale and Numeric Rating Scale for measuring pain intensity; (2) Roland-Morris Disability Questionnaire and Oswestry Disability Index for measuring disability; (3) 36-item Short Form Survey (SF-36), 12-item Short Form Survey (SF-12), and Global Perceived Recovery for measuring overall health status; (4) Patient Satisfaction Questionnaire for measuring satisfaction with care; and (5) National Ambulatory Medical Care Survey and 73-item LBP health care utilization questionnaire for measuring health care utilization. Associations at different outcome follow-up periods and all durations/periods of follow-up are eligible. Effect measures of interest include odds ratio or risk ratio for dichotomous data, rates or rate ratios for count data, mean differences for continuous data, and survival time or hazard rate ratios for time-to-event data. If not reported by the studies, these effect measures will be computed, when applicable, based on available data in the studies.

### Study designs/characteristics

Eligible studies targeting the population, exposure, and outcomes listed above must meet the following criteria: (1) English language (to increase feasibility) and (2) cohort or case-control studies. Studies that present secondary analyses of randomized trials (e.g., control group only) will be included. We will provide a list of possibly relevant titles in other languages in the final manuscript. The following will be excluded: (1) guidelines, letters, editorials, commentaries, books and book chapters, conference proceedings, meeting abstracts, lectures and addresses, and consensus development statements; (2) case reports, case series, cross-sectional studies, randomized controlled trials, qualitative studies, systematic and non-systematic reviews, biomechanical studies, laboratory studies, and studies not reporting on methodology; and (3) cadaveric or animal studies.

### Information sources and search strategy

MEDLINE, Embase, CINAHL, and PsycINFO will be searched from database inception to April 2019. The search strategy will be developed in consultation with an experienced health sciences librarian (see Additional file 2), which will be reviewed by a second librarian using the Peer Review of Electronic Search Strategies (PRESS) Checklist [60, 61]. The search strategy will be developed in MEDLINE and adapted to the other bibliographic databases. Search terms will include subject headings (e.g., MeSH in MEDLINE) for each database and free text words for the key concepts of LBP, psychosocial factors, and depressive symptoms/depression. EndNote will be used to de-duplicate references electronically across databases and record the number of duplicates identified.

The reference lists of included studies and related systematic reviews [22, 31–36] will be searched. Citation searching of questionnaires for depressive symptoms that have been utilized in LBP populations will also be conducted: Center for Epidemiologic Studies Depression Scale, Beck Depression Index, Patient Health Questionnaire-9, and Depression Scale of the Hospital Anxiety and Depression Scale.

### Data collection and analysis

#### Study selection

A two-phase (titles and abstracts; full-text articles) screening process will be used to select eligible studies. A training exercise will be conducted before starting screening to ensure reliability. Team members will screen a random sample of 50 records from the literature search based on titles and abstracts using the predefined inclusion and exclusion criteria. Team members will conduct a similar training exercise for screening potentially relevant full-text articles using a random sample of 25 full-text articles. Agreement of at least 80% for phase I (i.e., classifying articles as possibly relevant versus irrelevant) and phase II (i.e., relevant versus irrelevant) screening during the training exercise will be assessed. If agreement is below these thresholds, all team members will discuss to resolve disagreements and establish clarifications to the eligibility criteria if needed before starting screening. In phase I screening, pairs of independent reviewers (JJW, CYL, JL) will screen citation titles and abstracts to determine the eligibility of studies by categorizing studies as possibly relevant or irrelevant. Pairs of independent reviewers (JJW, CYL, JL) will screen possibly relevant studies in full text during phase II screening to determine eligibility and document reasons for exclusion. Reviewers will meet to discuss disagreements and reach consensus on the eligibility of studies by categorizing studies as relevant or irrelevant. A third reviewer (ACT, PC, or LR) will be involved if consensus cannot be reached. Study authors will be contacted for additional information as needed when screening, assessing risk of bias, and conducting data extraction of studies.

#### Data items and data collection process

Data extraction forms will be drafted and pilot tested. A training exercise will be conducted using a random sample of five articles to pilot test the standardized data abstraction form involving all reviewers and assess for at least 80% agreement before starting full data extraction. The lead author will extract data from eligible studies to build evidence tables. A second reviewer will independently extract study results (e.g., effect size, 95% CI) and any disagreements will be discussed to reach consensus. A second reviewer will verify all other data extraction items by checking the extracted data to minimize error.

Data will be extracted from each study on author, publication year, study design, setting and participants (age, sex, number at baseline and follow-up), duration of follow-up, definition of exposure and outcomes, comparison groups, effect sizes and 95% confidence intervals from unadjusted and adjusted analyses, and covariate information (see Additional file 3). Effect sizes include risk ratios, rate ratios, odds ratios, hazard ratios, and mean differences. Authors will be contacted if there is missing information in the studies, such as effect estimates or raw data. However, if this information is still missing after attempted contact, these study results will be described separately based on available information (e.g., when only statistical significance is reported as yes/no).

### Methodological quality and risk of bias appraisal

As a training exercise to ensure reliability, two reviewers will independently appraise a random subset of five included studies using all risk of bias appraisal components of the Risk of Bias in Non-randomized Studies—of Exposures (ROBINS-E) tool [62]. The ROBINS-E tool assesses seven domains of risk of bias related to confounding, selection of participants, classification of exposures, departures from intended exposures, missing data, measurement of outcomes, and selection of reported results. Reviewers will summarize judgments within each domain to assess the overall risk of bias for each study. Age and sex have been pre-specified as the minimal set of confounding in the risk of bias assessment using ROBINS-E. Any discrepancies will be resolved through discussion or by involving a third reviewer, and clarifications to ROBINS-E will be established if needed before starting the risk of bias assessment. All reviewers will be trained in the use of this critical appraisal instrument. Pairs of independent reviewers will critically appraise eligible studies using ROBINS-E. Paired reviewers will discuss disagreements to reach consensus, and a third reviewer will be involved if consensus cannot be reached. ROBINS-E tool will be used to evaluate the presence and impact of selection bias, information bias, and confounding on study results [62]. Reviewers will use these criteria to inform their judgment on the internal validity of studies (e.g., low, moderate, versus high risk of bias).

### Synthesis of included studies

The percent agreement will be computed for all stages of pilot testing and risk of bias assessment (i.e., agreement for classifying studies into low or high risk of bias). The percent agreement and kappa of agreement will be computed for all stages of screening and data extraction. Clinical, methodological, and statistical (using the *I*^2^ statistic) [63] heterogeneity among studies will be assessed. Clinical heterogeneity may result from differences in populations, exposures, comparators, or outcomes across studies. Methodological and statistical heterogeneity may result from differences in risk of bias and differences in outcomes across studies beyond what could be expected by chance alone. Methodological heterogeneity across studies will be assessed based on the overall judgment from ROBINS-E as low or moderate versus high risk of bias. Statistical heterogeneity will be assessed using the *I*^2^ statistic, whereby *I*^2^ of < 25–50% will be considered low to moderate (homogeneous), and ≥ 50% considered high (heterogeneous) [63].

A random effects meta-analysis will be performed on the association between depressive symptoms and health outcomes if studies are deemed homogeneous (Table 1). Specifically, a random effects meta-analysis will be conducted using the odds ratio or risk ratio effect measure for dichotomous data, rates or rate ratios for count data, mean differences for continuous data, and hazard rate ratios for time-to-event data when at least two studies are deemed homogeneous. Reported numbers from studies will be converted to rates by extracting the number of cases (numerator), population at risk, and follow-up time (denominator) if available. To explore the impact of methodological quality (risk of bias) on study results, the following meta-analyses as sensitivity analyses will be conducted: (1) including all studies (i.e., low, moderate, and high risk of bias studies) and (2) including low risk of bias studies only. If studies are deemed heterogeneous, the results of the included studies will be descriptively outlined, stratified by low/moderate versus high risk of bias studies.

**Table 1:** Categories to guide the assessment of homogeneity

Category	Description^a^
1A	Population: type of LBP - LBP without radiculopathy OR - LBP with radiculopathy
1B	Population: duration of LBP - Acute/subacute (< 12 weeks’ duration) OR - Chronic (≥ 12 weeks’ duration)
2A	Exposure: type of condition - Depressive symptoms OR - Depression
2B	Exposure: severity of condition - Mild (e.g., mild depression) OR - Severe (e.g., severe depression)
3	Outcome: type - Pain intensity - LBP-related disability - Health-related quality of life - Type of health care utilization (e.g., family physician visit, specialist visit, or spinal radiograph)

*LBP* low back pain

^a^Describes how studies within the listed categories would be considered homogeneous (e.g., studies targeting the following would be considered homogeneous: (1) chronic LBP without radiculopathy as the population, (2) severe depression as the exposure, and (3) pain intensity as the outcome)

Results will be further stratified by type of LBP (with or without radiculopathy; mixed populations with radiculopathy and no radiculopathy; versus unclear), duration of LBP (acute/subacute, i.e., < 3 months’ duration versus chronic, i.e., ≥ 3 months’ duration), type of exposure (depressive symptoms versus depression), and health outcome (e.g., pain intensity, disability). Analyses will be conducted separately for cohort and case-control studies, and for unadjusted and minimally adjusted associations (i.e., adjusted for age and sex). As recommended for prognostic factor systematic reviews, a meta-analysis will be conducted for hazard ratios, odds ratios, and risk ratios separately [64]. After stratifying results, outcomes that are reported in at least 10 studies in the meta-analysis will be assessed for publication bias by visually inspecting funnel plots for asymmetry [65, 66]. Results from studies that adjusted for the minimally required set of confounding will be focused on when interpreting results. Thresholds for minimal clinically important differences will be used to judge the clinical importance of outcomes where applicable: 10/100 mm for the Visual Analog Scale [67], 2/10 for the Numeric Rating Scale [68], 5/24 for the Roland-Morris Disability Questionnaire [69], 10/100 for the Oswestry Disability Index [69], 7.7 points for the Physical Component Summary, and 10 points for Bodily Pain on the Short-Form-36 [70]. The summary results will be interpreted by considering the direction, magnitude, and precision of effect estimates across studies, impact of risk of bias in sensitivity analyses, potential for publication bias, and generalizability of findings.

## Discussion and dissemination of results

Our systematic review will provide a comprehensive synthesis of the evidence to advance our understanding of the association between depressive symptoms/depression and health outcomes among adults with LBP. However, there are limitations and challenges to our proposed systematic review. Only studies in English will be included in the systematic review to increase feasibility. However, a previous study found no evidence of systematic bias when using language restrictions in systematic reviews with meta-analyses in conventional medicine [71]. We will provide a list of possibly relevant titles in other languages in the final manuscript. In addition, the existing literature varies greatly in the measures and indices used to assess health outcomes and recovery related to LBP. A number of approaches has been incorporated to overcome this challenge. First, the selected health outcomes of interest are informed by core outcome domains that international expert panels have deemed important for LBP research [57–59]. Only standardized outcome measures (e.g., standardized questionnaires or administrative data) will be included to streamline the data extraction and synthesis. Second, the parameters related to the population, exposure, comparator, and outcome (Table 1) that would be considered for homogeneity have been outlined. Third, a random effects meta-analysis will be performed on the association between depressive symptoms and health outcomes if studies are deemed homogeneous; however, the results of the included studies will be descriptively outlined if studies are deemed heterogeneous.

Our multifaceted knowledge translation and exchange strategy is tailored to the various stakeholders that would be interested in the findings of this systematic review. The results will be presented at scientific meetings and conferences focused on spine research (e.g., EUROSPINE) to disseminate results to the scientific community, including researchers and academics. The manuscript will be submitted to a relevant high impact, peer-reviewed journal (e.g., *Spine Journal*) and for open-access publication. In addition, a 1-page research brief will be drafted to be (1) posted on the website of the Knowledge Translation Program at St. Michael’s Hospital; (2) circulated to Knowledge Translation Canada, which reaches over 2000 researchers and knowledge users in Canada; and (3) circulated to the Strategy for Patient-Oriented Research (SPOR) Evidence Alliance, which reaches over 250 researchers and knowledge users in Canada and abroad. To engage with health care professionals and decision-makers, this research brief will also be circulated to Health Quality Ontario, Choosing Wisely Canada, and LBP models of care in Ontario, including the Inter-professional Spine Assessment and Education Clinics, and Primary Care Low Back Pain Pilot program. Finally, key messages will be posted through a Twitter campaign to disseminate results to the community and general public.

Overall, findings from our systematic review will be relevant to patients, health care providers, researchers, and decision-makers. Understanding the impact of depressive symptoms and depression is necessary to guide expectations and clinical management of LBP among patients and health care providers. Information about prognostic factors can help health care providers identify patients at risk of developing chronic LBP and disability. In turn, appropriate care and management of depressive symptoms and depression in this patient population may help improve LBP recovery. From a health system perspective, our research will help guide better resource allocation for health programs and strategies targeting key prognostic factors for LBP. Our systematic review will also identify key knowledge gaps related to depressive symptoms, depression, and LBP prognosis to inform future research directions. Ultimately, understanding the impact of depressive symptoms and depression on health outcomes for LBP will help tailor resources, health services delivery, and quality of care to improve health outcomes in adults with LBP.

## Supplementary information

**Additional file 1.** PRISMA-P 2015 Checklist. Preferred Reporting Items for Systematic Review and Meta-Analysis Protocols (PRISMA-P) 2015 Statement.

**Additional file 2.** Search Strategies.

**Additional file 3.** Data Extraction Form.

## Data Availability

Not applicable

## Abbreviations

LBP: Low back pain
PRISMA: Preferred Reporting Items for Systematic Review and Meta-analysis
PRISMA-P: Preferred Reporting Items for Systematic Review and Meta-analysis Protocols
