# Supplementary material for: The association between depressive symptoms or depression and health outcomes in adults with low back pain with or without radiculopathy: protocol of a systematic review
Source: Syst Rev. 2019 Nov 8;8:267. doi: 10.1186/s13643-019-1192-4 (PMC6839250; doi:10.1186/s13643-019-1192-4)
Supplement: Supplementary file 2 — Additional file 2. Search Strategies. [file 13643_2019_1192_MOESM2_ESM.pdf]

Additional File 2. Search Strategies

MEDLINE

Ovid MEDLINE(R) ALL <1946 to Present>

#	**Searches**
1	exp Back/
2	exp Back Injuries/
3	exp Back Muscles/
4	exp Back Pain/
5	Coccyx/
6	Discitis/
7	exp Intervertebral Disk/
8	Lumbar Vertebrae/
9	exp Lumbosacral Plexus/
10	Osteoarthritis/
11	Osteoarthritis, Spine/
12	Polyradiculopathy/
13	Radiculopathy/
14	Sacroiliac Joint/
15	Sacrum/
16	exp Sciatic Neuropathy/
17	exp Spinal Diseases/
18	Spine/
19	exp Spondylarthritis/
20	or/1-19
21	(back pain or backache or back ache or low* back pain*).ti,ab,kw,kf.
22	((backache* or back ache*) adj3 (injur* or pain*)).ti,ab,kw,kf.
23	((back or lumb*) adj3 (ache* or injur* or pain*)).ti,ab,kw,kf.
24	coccydynia.ti,ab,kw,kf.
25	((coccyx or coccygeal or (L1 adj4 L5) or lumbarsacr* or lumbosacr* or lumbar or sacral or sacrococcygeal or sacroiliac or sacro iliac or spinal or tailbone* or vertebrogenic) adj3 (ache* or break* or broke* or bruis* or discomfort or fractur* or hurt or injur* or pain* or tender* or trauma)).ti,ab,kw,kf.
26	((disc* or disk*) adj3 (avuls* or bulg* or compress* or extrude* or degenerat* or displac* or herniat* or hurt or injur* or pain* or protrud* or prolaps* or ruptur* or sequester* or slip* or trauma or tear? or torn)).ti,ab,kw,kf.
27	(discitides or discitis or diskitides or diskitis or spondylodiscitides or spondylodiscitis or spondylodiskitides or spondylodiskitis).ti,ab,kw,kf.
28	dorsalgia.ti,ab,kw,kf.
29	lumbago.ti,ab,kw,kf.
30	(lumbar adj (disc* or disk*) adj3 (avuls* or extrude* or degenerat* or displac* or herniat* or prolaps* or sequester* or slip* or injur* or pain*)).ti,ab,kw,kf.
31	(lumbar adj3 (compres* or facet* or nerve root* or osteoarthritis or radicul* or spinal stenos#s or spondylo* or zygapophys*)).ti,ab,kw,kf.
32	lumboischialgia.ti,ab,kw,kf.
33	Piriformis syndrome*.ti,ab,kw,kf.
34	radiculalgia.ti,ab,kw,kf.
35	sciatic*.ti,ab,kw,kf.
36	spondylosis.ti,ab,kw,kf.
37	((stenos#s adj (spine or root or spinal)) or (failed adj3 "back syndrome*")).ti,ab,kw,kf.

38	or/21-37
39	20 or 38
40	Adaptation, Psychological/
41	Affective Symptoms/
42	Anxiety/
43	Anxiety Disorders/
44	Depression/
45	Depressive Disorder/
46	Depressive Disorder, Major/
47	Dysthymic Disorder/
48	Irritable Mood/
49	Mental Health/
50	Mood Disorders/
51	Neurotic Disorders/
52	Sadness/
53	Stress, Psychological/
54	Suicidal Ideation/
55	Resilience, Psychological/
56	or/40-55
57	(depression or depressive or depressed).ti,kf,kw.
58	(depression or depressive or depressed).ab. /freq=2
59	((emotional* or mental* or psycholog*) adj3 (adapt* or affliction* or cope? or coping or distress* or health* or illness* or resilien* or stress* or unhealth* or unwell or well being or wellbeing or wellness)).ti,ab,kw,kf.
60	(anguish* or anxiety or anxious* or dejected or desolat* or despair* or desperat* or desponden* or distress* or dysthymi* or hopeless* or irritability or joyless* or melanchol* or mood* or misery or miserable or morose or sadness or self-despair* or self-pity* or unhap*).ti,kw,kf. or (anguish* or anxiety or anxious* or dejected or desolat* or despair* or desperat* or desponden* or distress* or dysthymi* or hopeless* or irritability or joyless* or melanchol* or mood* or misery or miserable or morose or sadness or self-despair* or self-pity* or unhap*).ab. /freq=2
61	(neuros#s or neurotic).ti,ab,kw,kf.
62	or/57-61
63	56 or 62
64	exp Case-Control Studies/
65	Control Groups/ or Matched-Pair Analysis/ or ((case* adj3 control*) or (case* adj3 comparison*) or control group*).ti,ab,kw.
66	exp Cohort Studies/
67	((cohort or longitudinal or prospective or retrospective) adj4 (study or studies or analy*)).ti,ab.
68	or/64-67
69	39 and 63 and 68
70	(exp Animals/ not Humans/) or ((murin* or mouse or mice or rat or rats or dog or dogs or cat or cats).ti. and ("in data review" or in process or publisher or "pubmed not medline").st.)
71	69 not 70
72	limit 71 to english

EMBASE

Ovid Embase <1974 to Present>

#	Searches

1	backache/
2	low back pain/
3	pelvic girdle pain/
4	discogenic pain/
5	cervical disk hernia/
6	diskitis/
7	exp experimental sciatic nerve injury/
8	failed back surgery syndrome/
9	exp intervertebral disk disease/
10	lumbar disk hernia/
11	lumbar plexus block/
12	lumbar spinal stenosis/
13	lumbar sympathectomy/
14	osteoarthritis/
15	exp radiculopathy/
16	sacroiliitis/
17	sciatic neuropathy/
18	exp sciatic nerve injury/
19	sciatica/
20	exp spine disease/
21	exp back/
22	exp pelvic girdle/
23	coccygeal vertebra/
24	coccygeus muscle/
25	coccyx/
26	exp intervertebral disk/
27	lumbar disk/
28	exp lumbar vertebra/
29	lumbar spine/
30	lumbosacral plexus/
31	lumbosacral spine/
32	sacroiliac joint/
33	sciatic nerve/
34	dislocation/
35	exp bone pain/
36	exp chronic pain/
37	exp inflammatory pain/
38	injury/
39	exp limb pain/
40	exp musculoskeletal pain/
41	pain/
42	phantom pain/
43	posttraumatic pain/
44	referred pain/
45	rupture/
46	spinal pain/
47	or/21-33
48	or/34-46
49	47 and 48
50	or/1-20
51	49 or 50

52	(back pain or backache or back ache or low* back pain*).ti,ab,kw.
53	((backache* or back ache*) adj3 (injur* or pain*)).ti,ab,kw.
54	((back or lumb*) adj3 (ache* or injur* or pain*)).ti,ab,kw.
55	coccydynia.ti,ab,kw.
56	((coccyx or coccygeal or (L1 adj4 L5) or lumbarsacr* or lumbosacr* or lumbar or sacral or sacrococcygeal or sacroiliac or sacro iliac or spinal or tailbone* or vertebrogenic) adj3 (ache* or break* or broke* or bruis* or discomfort or fractur* or hurt or injur* or pain* or tender* or trauma)).ti,ab,kw.
57	((disc* or disk*) adj3 (avuls* or bulg* or compress* or extrude* or degenerat* or displac* or herniat* or hurt or injur* or pain* or protrud* or prolaps* or ruptur* or sequester* or slip* or trauma or tear? or torn)).ti,ab,kw.
58	(discitides or discitis or diskitides or diskitis or spondylodiscitides or spondylodiscitis or spondylodiskitides or spondylodiskitis).ti,ab,kw.
59	lumbago.ti,ab,kw.
60	(lumbar adj (disc* or disk*) adj3 (avuls* or extrude* or degenerat* or displac* or herniat* or prolaps* or sequester* or slip* or injur* or pain*)).ti,ab,kw.
61	(lumbar adj3 (compres* or facet* or nerve root* or osteoarthritis or radicul* or spinal stenos#s or spondylo* or zygapophys*)).ti,ab,kw.
62	lumboischialgia.ti,ab,kw.
63	Piriformis syndrome*.ti,ab,kw.
64	radiculalgia.ti,ab,kw.
65	sciatic*.ti,ab,kw.
66	spondylosis.ti,ab,kw.
67	((stenos#s adj (spine or root or spinal)) or (failed adj3 "back syndrome*")).ti,ab,kw.
68	or/52-67
69	51 or 68
70	affective neurosis/
71	anxiety/
72	anxiety disorder/
73	anxiety neurosis/
74	exp depression/
75	exp depression assessment/
76	distress syndrome/
77	emotional stress/
78	major affective disorder/
79	minor affective disorder/
80	mental health/
81	"mixed anxiety and depression"/
82	mood change/
83	mood disorder/
84	neurosis/
85	psychological resilience/
86	sadness/
87	suicidal ideation/
88	or/70-87
89	(depression or depressive or depressed).ti,kw.
90	(depression or depressive or depressed).ab. /freq=2
91	((emotional* or mental* or psycholog*) adj3 (adapt* or affliction* or cope? or coping or distress* or health* or illness* or resilien* or stress* or unhealth* or unwell or well being or wellbeing or wellness)).ti,ab,kw.

92	(anguish* or anxiety or anxious* or dejected or desolat* or despair* or desperat* or desponden* or distress* or dysthymi* or hopeless* or irritability or joyless* or melanchol* or mood* or misery or miserable or morose or sadness or self-despair* or self-pity* or unhap*).ti,kw. or (anguish* or anxiety or anxious* or dejected or desolat* or despair* or desperat* or desponden* or distress* or dysthymi* or hopeless* or irritability or joyless* or melanchol* or mood* or misery or miserable or morose or sadness or self-despair* or self-pity* or unhap*).ab. /freq=2
93	(neuros#s or neurotic).ti,ab,kw.
94	or/89-93
95	88 or 94
96	69 and 95
97	exp case control study/
98	controlled study/ or pretest posttest control group design/ or static group comparison/ or ((case* adj3 control*) or (case* adj3 comparison*) or control group*).ti,ab,kw.
99	cohort analysis/
100	((cohort or longitudinal or prospective or retrospective) adj4 (study or studies or analy*)).ti,ab.
101	or/97-100
102	96 and 101
103	(exp animal/ or animal experiment/ or nonhuman/) not exp human/
104	102 not 103

CINAHL

EBSCOhost CINAHL Plus with Full Text

#	Query
S1	(MH "Back")
S2	(MH "Back Injuries+")
S3	(MH "Back Pain+")
S4	(MH "Coccyx")
S5	(MH "Discitis")
S6	(MH "Intervertebral Disk+")
S7	(MH "Lumbar Vertebrae")
S8	(MH "Lumbosacral Plexus+")
S9	(MH "Osteoarthritis, Spine+")
S10	(MH "Piriformis Syndrome")
S11	(MH "Radiculopathy")
S12	(MH "Polyradiculopathy")
S13	(MH "Sacroiliac Joint")
S14	(MH "Sacroiliac Joint Dysfunction")
S15	(MH "Sacrum")
S16	(MH "Sciatic Nerve+")
S17	(MH "Sciatica")
S18	(MH "Spinal Diseases+")
S19	(MH "Spinal Injuries+")
S20	(MH "Spine")
S21	S1 OR S2 OR S3 OR S4 OR S5 OR S6 OR S7 OR S8 OR S9 OR S10 OR S11 OR S12 OR S13 OR S14 OR S15 OR S16 OR S17 OR S18 OR S19 OR S20
S22	TI (("back pain" OR backache OR "back ache" OR "low* back pain")) OR AB (("back pain" OR backache OR "back ache" OR "low* back pain")) OR SU (("back pain" OR backache OR "back ache" OR "low* back pain"))

S23	TI (((backache* OR "back ache*") N3 (injur* OR pain*))) OR AB (((backache* OR "back ache*") N3 (injur* OR pain*))) OR SU (((backache* OR "back ache*") N3 (injur* OR pain*)))
S24	TI (((back OR lumb*) N3 (ache* OR injur* OR pain*))) OR AB (((back OR lumb*) N3 (ache* OR injur* OR pain*))) OR SU (((back OR lumb*) N3 (ache* OR injur* OR pain*)))
S25	TI coccydynia OR AB coccydynia OR SU coccydynia
S26	TI (((coccyx OR coccygeal OR (L1 N4 L5) OR lumbarsacr* OR lumbosacr* OR lumbar OR sacral OR sacrococcygeal OR sacroiliac OR sacro iliac OR spinal OR tailbone* OR vertebrogenic) N3 (ache* OR break* OR broke* OR bruis* OR discomfort OR fractur* OR hurt OR injur* OR pain* OR tender* OR trauma))) OR AB (((coccyx OR coccygeal OR (L1 N4 L5) OR lumbarsacr* OR lumbosacr* OR lumbar OR sacral OR sacrococcygeal OR sacroiliac OR sacro iliac OR spinal OR tailbone* OR vertebrogenic) N3 (ache* OR break* OR broke* OR bruis* OR discomfort OR fractur* OR hurt OR injur* OR pain* OR tender* OR trauma))) OR SU (((coccyx OR coccygeal OR (L1 N4 L5) OR lumbarsacr* OR lumbosacr* OR lumbar OR sacral OR sacrococcygeal OR sacroiliac OR sacro iliac OR spinal OR tailbone* OR vertebrogenic) N3 (ache* OR break* OR broke* OR bruis* OR discomfort OR fractur* OR hurt OR injur* OR pain* OR tender* OR trauma)))
S27	TI (((disc* OR disk*) N3 (avuls* OR bulg* OR compress* OR extrude* OR degenerat* OR displac* OR herniat* OR hurt OR injur* OR pain* OR protrud* OR prolaps* OR ruptur* OR sequester* OR slip* OR trauma OR tear# OR torn))) OR AB (((disc* OR disk*) N3 (avuls* OR bulg* OR compress* OR extrude* OR degenerat* OR displac* OR herniat* OR hurt OR injur* OR pain* OR protrud* OR prolaps* OR ruptur* OR sequester* OR slip* OR trauma OR tear# OR torn))) OR SU (((disc* OR disk*) N3 (avuls* OR bulg* OR compress* OR extrude* OR degenerat* OR displac* OR herniat* OR hurt OR injur* OR pain* OR protrud* OR prolaps* OR ruptur* OR sequester* OR slip* OR trauma OR tear# OR torn)))
S28	(discitides OR discitis OR diskitides OR diskitis OR spondylodiscitides OR spondylodiscitis OR spondylodiskitides OR spondylodiskitis)
S29	TI dorsalgia OR AB dorsalgia OR SU dorsalgia
S30	TI lumbago OR AB lumbago OR SU lumbago
S31	TI ((lumbar N1 (disc* OR disk*) N3 (avuls* OR extrude* OR degenerat* OR displac* OR herniat* OR prolaps* OR sequester* OR slip* OR injur* OR pain*))) OR AB ((lumbar N1 (disc* OR disk*) N3 (avuls* OR extrude* OR degenerat* OR displac* OR herniat* OR prolaps* OR sequester* OR slip* OR injur* OR pain*))) OR SU ((lumbar N1 (disc* OR disk*) N3 (avuls* OR extrude* OR degenerat* OR displac* OR herniat* OR prolaps* OR sequester* OR slip* OR injur* OR pain*)))
S32	TI ((lumbar N3 (compres* OR facet* OR nerve root* OR osteoarthritis OR radicul* OR spinal stenos?s OR spondylo* OR zygapophys*))) OR AB ((lumbar N3 (compres* OR facet* OR nerve root* OR osteoarthritis OR radicul* OR spinal stenos?s OR spondylo* OR zygapophys*))) OR SU ((lumbar N3 (compres* OR facet* OR nerve root* OR osteoarthritis OR radicul* OR spinal stenos?s OR spondylo* OR zygapophys*)))
S33	TI lumboischialgia OR AB lumboischialgia OR SU lumboischialgia
S34	TI "Piriformis syndrome*" OR AB "Piriformis syndrome*" OR SU "Piriformis syndrome*"
S35	TI radiculalgia OR AB radiculalgia OR SU radiculalgia
S36	TI sciatic* OR AB sciatic* OR SU sciatic*
S37	TI spondylosis OR AB spondylosis OR SU spondylosis
S38	TI (((stenos?s N1 (spine OR root OR spinal)) OR (failed N3 "back syndrome*"))) OR AB (((stenos?s N1 (spine OR root OR spinal)) OR (failed N3 "back syndrome*"))) OR SU (((stenos?s N1 (spine OR root OR spinal)) OR (failed N3 "back syndrome*")))
S39	S22 OR S23 OR S24 OR S25 OR S26 OR S27 OR S28 OR S29 OR S30 OR S31 OR S32 OR S33 OR S34 OR S35 OR S36 OR S37 OR S38
S40	S21 OR S39

S41	(MH "Adaptation, Psychological")
S42	(MH "Adjustment Disorders")
S43	(MH "Affective Disorders")
S44	(MH "Affective Symptoms")
S45	(MH "Anxiety")
S46	(MH "Anxiety Disorders")
S47	(MH "Depression")
S48	(MH "Depression, Reactive")
S49	(MH "Dysthymic Disorder")
S50	(MH "Hardiness")
S51	(MH "Mental Health")
S52	(MH "Neurotic Disorders")
S53	(MH "Sadness")
S54	(MH "Stress, Psychological")
S55	(MH "Suicidal Ideation")
S56	S41 OR S42 OR S43 OR S44 OR S45 OR S46 OR S47 OR S48 OR S49 OR S50 OR S51 OR S52 OR S53 OR S54 OR S55
S57	TI ((depression OR depressive OR depressed)) OR SU ((depression OR depressive OR depressed))
S58	AB (depression OR depressive OR depressed)
S59	S58 OR S57
S60	TI (((emotional* OR mental* OR psycholog*) N3 (adapt* OR affliction* OR cope# OR coping OR distress* OR health* OR illness* OR resilien* OR stress* OR unhealth* OR unwell OR "well being" OR wellbeing OR wellness))) OR AB (((emotional* OR mental* OR psycholog*) N3 (adapt* OR affliction* OR cope# OR coping OR distress* OR health* OR illness* OR resilien* OR stress* OR unhealth* OR unwell OR "well being" OR wellbeing OR wellness))) OR SU (((emotional* OR mental* OR psycholog*) N3 (adapt* OR affliction* OR cope# OR coping OR distress* OR health* OR illness* OR resilien* OR stress* OR unhealth* OR unwell OR "well being" OR wellbeing OR wellness)))
S61	TI ((anguish* OR anxiety OR anxious* OR dejected OR desolat* OR despair* OR desperat* OR desponden* OR distress* OR dysthymi* OR hopeless* OR irritability OR joyless* OR melanchol* OR mood* OR misery OR miserable OR morose OR sadness OR self-despair* OR self-pity* OR unhap*)) OR SU ((anguish* OR anxiety OR anxious* OR dejected OR desolat* OR despair* OR desperat* OR desponden* OR distress* OR dysthymi* OR hopeless* OR irritability OR joyless* OR melanchol* OR mood* OR misery OR miserable OR morose OR sadness OR self-despair* OR self-pity* OR unhap*))
S62	AB (anguish* OR anxiety OR anxious* OR dejected OR desolat* OR despair* OR desperat* OR desponden* OR distress* OR dysthymi* OR hopeless* OR irritability OR joyless* OR melanchol* OR mood* OR misery OR miserable OR morose OR sadness OR self-despair* OR self-pity* OR unhap*)
S63	S61 OR S62
S64	TI ((neuros?s OR neurotic)) OR AB ((neuros?s OR neurotic)) OR SU ((neuros?s OR neurotic))
S65	S59 OR S60 OR S63 OR S64
S66	S56 OR S65
S67	(MH "Case Control Studies")
S68	(MH "Hospital-Based Case Control")
S69	(MH "Matched Case Control")
S70	(MH "Population-Based Case Control")
S71	(MH "Prospective Studies+")
S72	(MH "Panel Studies+")

S73	(MH "Pseudolongitudinal Studies")
S74	(MH "Retrospective Design")
S75	TI (((case* N3 control*) OR (case* N3 comparison*) OR control group*)) OR SU (((case* N3 control*) OR (case* N3 comparison*) OR control group*))
S76	S67 OR S68 OR S69 OR S70 OR S71 OR S72 OR S73 OR S74 OR S75
S77	S40 AND S66 AND S76
S78	S77 NOT ((MH "Animals+") NOT (MH "Human"))

PSYCINFO

Ovid PsycINFO <1806 to Present>

#	**Searches**
1	"back (anatomy)"/
2	back pain/
3	lumbar spinal cord/
4	musculoskeletal system/
5	neuropathic pain/
6	spinal column/
7	spinal nerves/
8	or/1-7
9	(back pain or backache or back ache or low* back pain*).ti,ab,id.
10	((backache* or back ache*) adj3 (injur* or pain*)).ti,ab,id.
11	((back or lumb*) adj3 (ache* or injur* or pain*)).ti,ab,id.
12	coccydynia.ti,ab,id.
13	((coccyx or coccygeal or (L1 adj4 L5) or lumbarsacr* or lumbosacr* or lumbar or sacral or sacrococcygeal or sacroiliac or sacro iliac or spinal or tailbone* or vertebrogenic) adj3 (ache* or break* or broke* or bruis* or discomfort or fractur* or hurt or injur* or pain* or tender* or trauma)).ti,ab,id.
14	((disc* or disk*) adj3 (avuls* or bulg* or compress* or extrude* or degenerat* or displac* or herniat* or hurt or injur* or pain* or protrud* or prolaps* or ruptur* or sequester* or slip* or trauma or tear? or torn)).ti,ab,id.
15	(discitides or discitis or diskitides or diskitis or spondylodiscitides or spondylodiscitis or spondylodiskitides or spondylodiskitis).ti,ab,id.
16	dorsalgia.ti,ab,id.
17	lumbago.ti,ab,id.
18	(lumbar adj (disc* or disk*) adj3 (avuls* or extrude* or degenerat* or displac* or herniat* or prolaps* or sequester* or slip* or injur* or pain*)).ti,ab,id.
19	(lumbar adj3 (compres* or facet* or nerve root* or osteoarthritis or radicul* or spinal stenos#s or spondylo* or zygapophys*)).ti,ab,id.
20	lumboischialgia.ti,ab,id.
21	Piriformis syndrome*.ti,ab,id.
22	radiculalgia.ti,ab,id.
23	sciatic*.ti,ab,id.
24	spondylosis.ti,ab,id.
25	((stenos#s adj (spine or root or spinal)) or (failed adj3 "back syndrome*")).ti,ab,id.
26	or/9-25
27	8 or 26
28	adjustment/
29	adjustment disorders/
30	anxiety/

31	anxiety disorders/
32	"depression (emotion)"/
33	dysthymic disorder/
34	emotional adjustment/
35	emotional states/
36	irritability/
37	major depression/
38	mental health/
39	neurosis/
40	psychological endurance/
41	reactive depression/
42	"resilience (psychological)"/
43	sadness/
44	stress reactions/
45	suicidal ideation/
46	or/28-45
47	(depression or depressive or depressed).ti,ab,id.
48	((emotional* or mental* or psycholog*) adj3 (adapt* or affliction* or cope? or coping or distress* or health* or illness* or resilien* or stress* or unhealth* or unwell or well being or wellbeing or wellness)).ti,id. or ((emotional* or mental* or psycholog*) adj3 (adapt* or affliction* or cope? or coping or distress* or health* or illness* or resilien* or stress* or unhealth* or unwell or well being or wellbeing or wellness)).ab. /freq=2
49	(anguish* or anxiety or anxious* or dejected or desolat* or despair* or desperat* or desponden* or distress* or dysthymi* or hopeless* or irritability or joyless* or melanchol* or mood* or misery or miserable or morose or sadness or self-despair* or self-pity* or unhap*).ti,id. or (anguish* or anxiety or anxious* or dejected or desolat* or despair* or desperat* or desponden* or distress* or dysthymi* or hopeless* or irritability or joyless* or melanchol* or mood* or misery or miserable or morose or sadness or self-despair* or self-pity* or unhap*).ab. /freq=2
50	(neuros#s or neurotic).ti,ab,id.
51	or/47-50
52	46 or 51
53	27 and 52
54	case control stud*.mp.
55	experiment controls/
56	longitudinal studies/ or prospective studies/ or retrospective studies/
57	((case* adj3 control*) or (case* adj3 comparison*) or control group*).ti,ab,id.
58	((cohort or longitudinal or prospective or retrospective) adj4 (study or studies or analy*)).ti,ab.
59	cohort analysis/
60	or/54-59
61	53 and 60
62	limit 61 to human
