# Supplementary material for: The association between depressive symptoms or depression and health outcomes in adults with low back pain with or without radiculopathy: protocol of a systematic review
Source: Syst Rev. 2019 Nov 8;8:267. doi: 10.1186/s13643-019-1192-4 (PMC6839250; doi:10.1186/s13643-019-1192-4)
Supplement: Supplementary file 3 — Additional file 3. Data Extraction Form. [file 13643_2019_1192_MOESM3_ESM.pdf]

Additional File 3. Data Extraction Form

						Definition of exposure/prognostic factor		Definition of outcome								Unadjusted analyses		Adjusted analyses		Covariates in adjusted analyses	
Author, publication year	Study design (cohort or case-control study)	Setting, country	# of participants at baseline	Age*	% F	Depressive symptoms **	Diagnosed depression* **	Pain	Disability	Overall health status	Satisfaction with care	Health care utilization	Comparison group definition	Follow-up (years)	Loss to follow-up (n, interval)	Effect size (risk ratio, rate ratio, OR, HRR, mean differ-ence)	95% CI	Effect size (risk ratio, rate ratio, OR, HRR, mean differ-ence)	95% CI	Minimal set (age, sex)	Additional covariates

CI – confidence interval; F – female; HRR – hazard rate ratio; OR – odds ratio

*Median, interquartile range or mean, standard deviation

**Center for Epidemiologic Studies Depression Scale, Beck Depression Index, Patient Health Questionnaire, or Depression Scale of the Hospital Anxiety and Depression Scale

***Diagnosed depression (major depressive disorder/episode or dysthymia) or self-reported diagnosis of depression
